# Brazil's nutrition labeling regulation: Challenges ahead on the path to guaranteeing consumer's right to adequate information

**DOI:** 10.3389/fnut.2022.921519

**Published:** 2022-11-22

**Authors:** Laís Amaral Mais, Camila Aparecida Borges, Neha Khandpur, Ana Clara Duran, Ana Paula Bortoletto Martins

**Affiliations:** ^1^Brazilian Institute for Consumer Defense (Idec), São Paulo, Brazil; ^2^Center for Epidemiological Studies for Nutrition and Health, University of São Paulo (Nupens, USP), São Paulo, Brazil; ^3^Center for Food Studies and Research, University of Campinas (NEPA, Unicamp), Campinas, Brazil; ^4^Department of Nutrition, University of São Paulo (USP), São Paulo, Brazil; ^5^Department of Nutrition, Harvard T. H. Chan School of Public Health, Boston, MA, United States

**Keywords:** front-of-package nutrition labeling, nutrition labeling, food regulation, nutrient profile model, warning label

## Introduction

Latin America and the Caribbean (LAC) face rising rates of multiple forms of malnutrition (1). Between 2019 and 2020, the prevalence of undernutrition increased in the region, with the COVID-19 pandemic aggravating pre-existing drivers of food and nutrition insecurity (2). At the same time, adult and childhood obesity rates remain alarming, with prevalences of almost 25 and 5%, respectively (3).

National governments have approved laws to improve the food environment and promote healthy diets, including the implementation of warning labels (WLs) as their front-of-package nutrition labels (FoPNL) to support healthier food choices. Over the last decade, in LAC countries, mandatory FoPNL using WLs have been implemented in Chile (4), Peru (5), Uruguay (6), Mexico (7) and Argentina (8). This type of mandatory FoPNL has been shown to lead to greater shifts in consumer food purchase intentions as compared with other FoPNL systems such as “traffic lights” (9–13).

In Brazil, the regulatory process to change the nutrition food labeling regulation and include a FoPNL, was started in 2014 by the National Health Surveillance Agency (*Agência Nacional de Vigilância Sanitária*–Anvisa). Anvisa is linked to the Ministry of Health (Executive power) and responsible for regulating the food labeling in the country *via* resolutions (14, 15). The regulatory process had the active participation of the government, the academia, civil society organizations and food and beverage industry representatives. After technical discussions in a working group, submission of proposals to support improvements in the current regulation, revision of international experiences and scientific evidence, and public consultations, the regulatory process was concluded in October 2020. The final regulation aimed to help consumers make more informed food purchase choices by: (i) including a mandatory FoPNL in the format of a magnifying glass to highlight excess added sugar, saturated fat and sodium in products where these nutrients were added; (ii) establishing new guidelines for the format, content, and legibility of the nutrition facts panel; and (iii) restricting nutrition claims on foods and beverages that would receive a FoPNL. According to Anvisa's resolution about nutrition labeling on packaged foods, nutrition labeling is “any statement intended to inform the consumer of the nutritional properties of the food, including the nutrition facts panel, FoPNL and nutrition claims”; and FoPNL is a “simplified standardized statement of high content of specific nutrients on the main panel of the food label” (14).

The regulatory process, however, was influenced by the food and beverage industry and their attempts to delay Anvisa's decision, derail the process, influence consumer opinion, and weaken the approved regulation (16). In this commentary, we present an overview of the changes incorporated to the nutrition labeling regulation in Brazil and highlight the strengths, limitations and potential challenges of the approved regulation.

## Changes to the FoPNL during the regulatory process

Despite several changes made to the FoPNL design and nutrient profile model (NPM) during the regulatory process (2014–2020), the available scientific evidence, that was free from conflict of interest and presented consistent and compelling evidence from Brazil and other LAC countries, was not fully incorporated, resulting in a final nutrition labeling regulation that could have done more to safeguard public health. This is likely because of the food industry's corporate political activities used throughout the process to weaken the technical discussions and to delay the approval of the regulation. Some examples of the discursive strategy are related to the loss of jobs and damage to the economy, the need for nutrition education with the focus on individual responsibility, the multifactorial cause of obesity promoting physical activity, balanced diets, smaller food portions and food reformulation, and against the Nanny-State (16). Regarding instrumental strategies, the food and beverage industry built the *Rede Rotulagem* (Labeling Network, in English) coalition, lobbied several decision makers, financed polls and research, and used legal action when it was opportune (17).

From 2014 to 2016, Anvisa coordinated a working group with government, academia, civil society and food industry representatives to discuss possible solutions to strengthen nutrition food labeling for packaged foods (18). In 2017, Anvisa received proposals for improving nutrition food labeling and, at the end of the same year, the official regulatory process was opened (19), based on the regulatory impact analysis (*análise de impacto regulatório*–AIR). The AIR is “a systematic evidence-based regulatory management process that seeks to assess, based on the definition of a regulatory problem, the possible impacts of the options available to achieve the intended objectives” (20). The AIR is run by Anvisa's technical team and incorporates two online public consultations: a preliminary technical consultation, which was aimed to base Anvisa's decisions and the draft of the regulation, and a final consultation, which invited general public feedback on the draft regulation.

**Figure 1:**
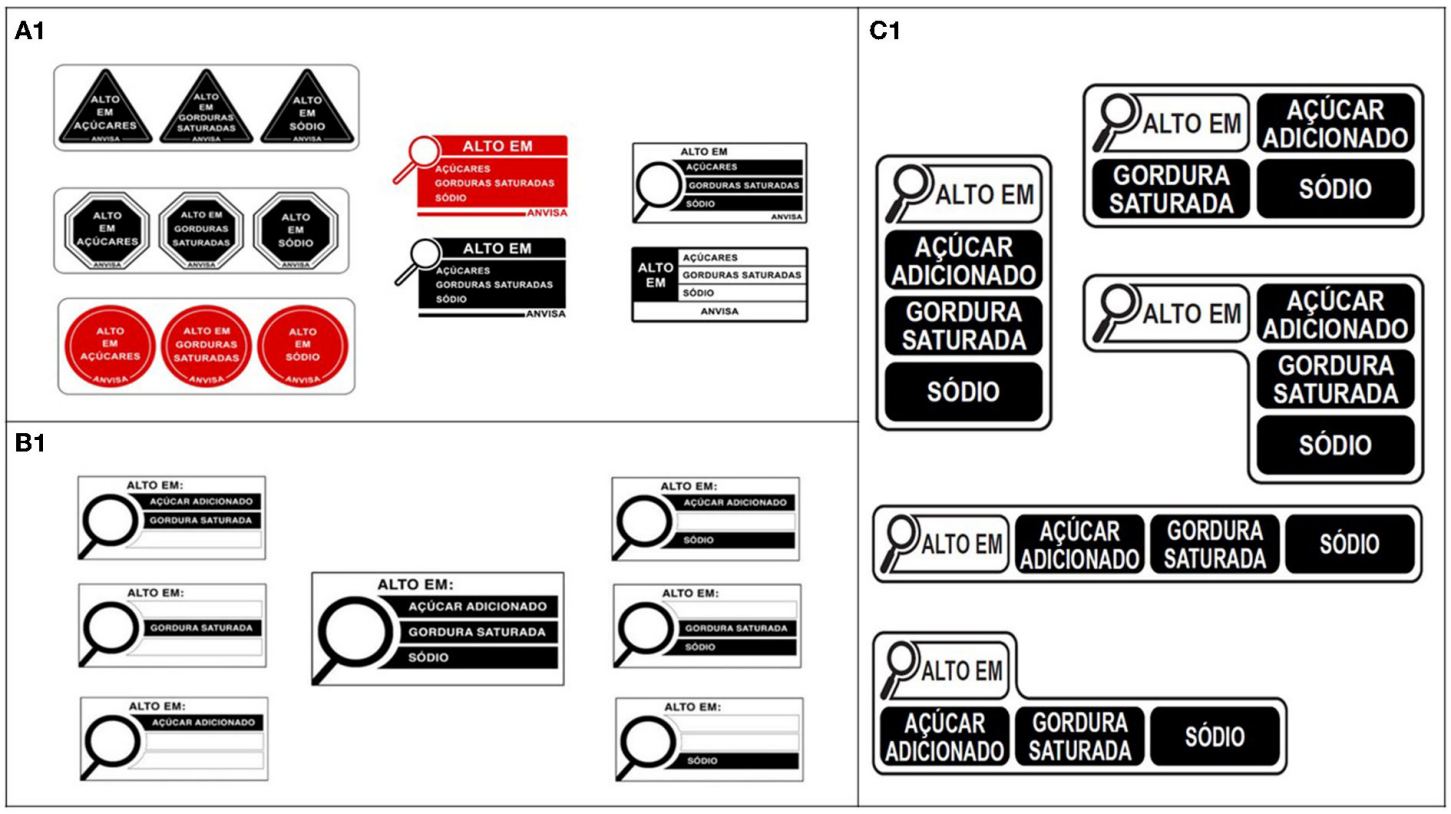
**(A1)** Design options of the FoPNL presented by Anvisa in the Preliminary Regulatory Impact Analysis Report on Nutrition Food Labeling in 2018. **(B1)** Design options of the FoPNL presented by Anvisa in the final public consultation in 2019. **(C1)** Final design of the FoPNL approved by Anvisa in 2020.

Anvisa's first technical proposal was presented in the “Preliminary Regulatory Impact Analysis Report on Nutrition Food Labeling” in 2018 (21). The report defined the regulatory objectives and identified that the best option for the FoPNL was a “high in” model that focused on excessive amounts of nutrients of concern that increase the risk of obesity and non-communicable diseases (NCDs). The NPM developed by Anvisa was based on the World Health Organization (WHO) (22) and the Codex Alimentarius (23) recommendations, with two gradual thresholds, starting with the less restrictive one. A variety of “high in” design options were presented for the FoPNL model (Figure 1A).

Anvisa's second technical proposal in 2019 presented the draft of the nutrition labeling regulation for public consultation. It included a FoPNL model in the format of a magnifying glass that would indicate high amounts of only three nutrients: added sugar, saturated fat and sodium (Figure 1B) (24, 25). The NPM would be implemented in two phases: less restrictive in the first year, and more restrictive after 2 years of implementation.

The final nutrition labeling regulation, which was approved by Anvisa's directors in 2020 based on the work of the technical team, presented a FoPNL with a revised magnifying glass design (14, 15) (Figure 1C), and a NPM with nutrient thresholds that had been previously proposed by Anvisa as an intermediate step, as follows: added sugar: ≥15/100 g; ≥7.5/100 ml; saturated fat: ≥6/100 g; ≥3/100 ml; sodium: ≥600 mg/100g; ≥300 mg/100 ml (thresholds for solid and semi-solid/liquid, respectively).

## Strengths and limitations of the Brazilian nutrition labeling regulation

The approved nutrition labeling regulation coherently targets the various nutrition information features available on food packages sold in Brazil, and has positive aspects worth highlighting. The nutrition facts panel had three main improvements. The first is the inclusion of mandatory information on total and added sugars, a recommendation by the WHO (26) to help consumers make more informed decisions as regards to nutrients that are associated with the development of obesity and diet-related NCDs. Second, food and beverage packages will be required to carry information on the content of nutrients by 100 g/ml. The same numerical base allows consumers to better compare between products. Nutrition content, currently presented by portion and percentage of daily value (%DV), are not necessarily based on each individual's consumption patterns and do not allow comparison between products from different food groups (27). The %DV is calculated for a pre-established 2,000-calorie diet for a healthy individual, which does not account for various nutritional requirements (28). Finally, the regulation proposes several design changes to improve legibility, optimizing color contrast with black writing on a white background, setting a minimum font size, standardizing the type of the font, as well as the placement of the nutrition facts panel on the food package.

The regulation also establishes strong and clear criteria for products to be targeted by the FoPNL and excludes unprocessed and minimally processed foods, selected processed foods (fresh fruits and vegetables, yogurt and other fermented milk beverages without added sugars, processed cheeses) with a low content of added sugars, sodium and saturated fat, breastmilk substitutes, nutrition supplements, and alcoholic beverages. These criteria align with the Brazilian Dietary Guidelines (29), as foods that are recommended for a healthy diet will not receive a “high in” label (30–32).

All products that exceed nutrient thresholds will have to mandatorily display a black and white “high in” FoPNL model, placed on the top half of the front panel of the food package. The color contrast and the position in the package improves label saliency, drawing consumer attention to the most important information (33, 34) and follows best practices of information design (35). The regulation forbids the use of other FoPNL models on the label. Nutrition claims on the package will also be restricted, but only for added sugars, fats and sodium if the product carries a FoPNL for these nutrients.

The regulation, however, falls short on several fronts. The design of the FoPNL–the magnifying glass–has not been implemented in any country so far. This model was first proposed in Canada, but was shown to be less effective than other tested options (36). In fact, the magnifying glass design incorporated in the Brazilian regulation had not even been tested prior to publication of the regulation in 2020. To our knowledge, only one study tested this design, published in 2021, and showed that the WLs (in the format of an octagon) were more effective than the magnifying glass in identifying the least harmful option, understanding the nutrient content, and shifting purchase intentions (37). The available evidence on the magnifying glass design presented during the public consultation showed better results for warning labels, such as octagons and triangles, on outcomes like time to detect the label, objective understanding of the nutritional content, perception of healthiness and purchase intention (38, 39). One study showed that the magnifying glass performed marginally better at improving purchase intentions than the triangles, despite having scored worse for objective understanding (40).

Regardless of the number of nutrients of concern in excess in the product, there will be only one magnifying glass printed on the package. This may be a better use of space in the package; however, the design does not benefit from having individual labels like the WLs, that catch the attention of the consumers and repeatedly alert them to excess nutrients (41). Also, the size specifications of the FoPNL may not be adequate relative to the size of the food packages, with smaller labels occupying less space on food labels, which may hinder the consumer's ability to notice them (42, 43).

The NPM and nutrients thresholds adopted in the approved nutrition labeling regulation have not been previously validated and have been shown to capture a lower proportion of unhealthy foods (as defined by the Nova classification system) (44) compared to currently implemented NPMs, such as the Pan-American Health Organization (PAHO) NPM and the Chilean NPM. The PAHO NPM identifies more foods high in nutrients of concern (62%) such as sweetened dairy and non-dairy beverages, canned vegetables, and convenience foods (45). Not to mention that with the adoption of the nutrient thresholds proposed by Anvisa, many ultra-processed foods and beverages will not receive a FoPNL, and will not contribute to help consumers overcome information barriers to follow the Brazilian Dietary Guidelines that recommends this type of food to be avoided (29).

The FoPNL in the approved nutrition labeling regulation only targets added sugar, saturated fat and sodium, leaving out other ingredients and nutrients of concern, such as low-calorie sweeteners (LCSs), trans fatty acids and caffeine. Recent, yet growing evidence, shows that LCSs are associated with higher risk of dysbiosis (46), abdominal obesity (47), non-communicable diseases (48) and metabolic abnormalities (49) and type 2 diabetes (50) in adults. For children, available evidence on the safety and effectiveness of consuming foods and beverages with LCSs is inconclusive (51). In fact, the consumption of foods and beverages with LCSs has been shown to increase their risk of developing NCDs as adults (48, 52). Consuming foods and beverages with LCSs may also lead to long-term and heightening of sweet taste preferences (53, 54). LCSs were found as an additive in 9% of all packaged foods and beverages sold in Brazil, in 15% of ultra-processed products, including in those foods and beverages with advertising on the FoPNL that targets children (55). In the case of trans fatty acids, their consumption has no known “safe level” and is related to increased risk of cardiovascular diseases, especially coronary heart disease (56–58), and mortality. WHO recommends the elimination of industrially produced trans fat. Limited data on the safety of consuming caffeine in sensitive population such as children and adolescents are available (59, 60). In the Mexican FoPNL regulation, all products with caffeine need to display a precautionary warning highlighting the presence of caffeine and that the product should be avoided by children (7).

The regulation also fails to ban all claims on products that will receive FoPNL, other than those related to the nutrients of the FoPNL. This may still leave consumers vulnerable to being misled by a potential “halo effect” of the presence of nutrition claims for other nutrients such as ‘high in fiber, vitamins and minerals' (61–64). In Brazil, claims are found in 41% of packaged products. Almost a third (28%) of packaged foods and beverages sold in the country carry a nutrition claim (e.g., source of calcium, 25% less sodium) and 22% carry a health claim (e.g., gluten free, natural). Importantly, foods with nutrition claims were more likely to be high in added sugar, sodium and/or saturated fat than those that would not receive a FoPNL (61).

The Brazilian approved nutrition labeling regulation does not include any restrictions on marketing targeted to children (like children's characters, cartoons, games, collectible gifts etc.) in foods high in nutrients of concern, as adopted in Chile and Mexico. Such marketing strategies are found in 20% of packaged foods and beverages sold in Brazil (55) and are known to increase children's desire for the product (65), brand loyalty (66) and demand for purchasing the product (67).

**Figure 2:**
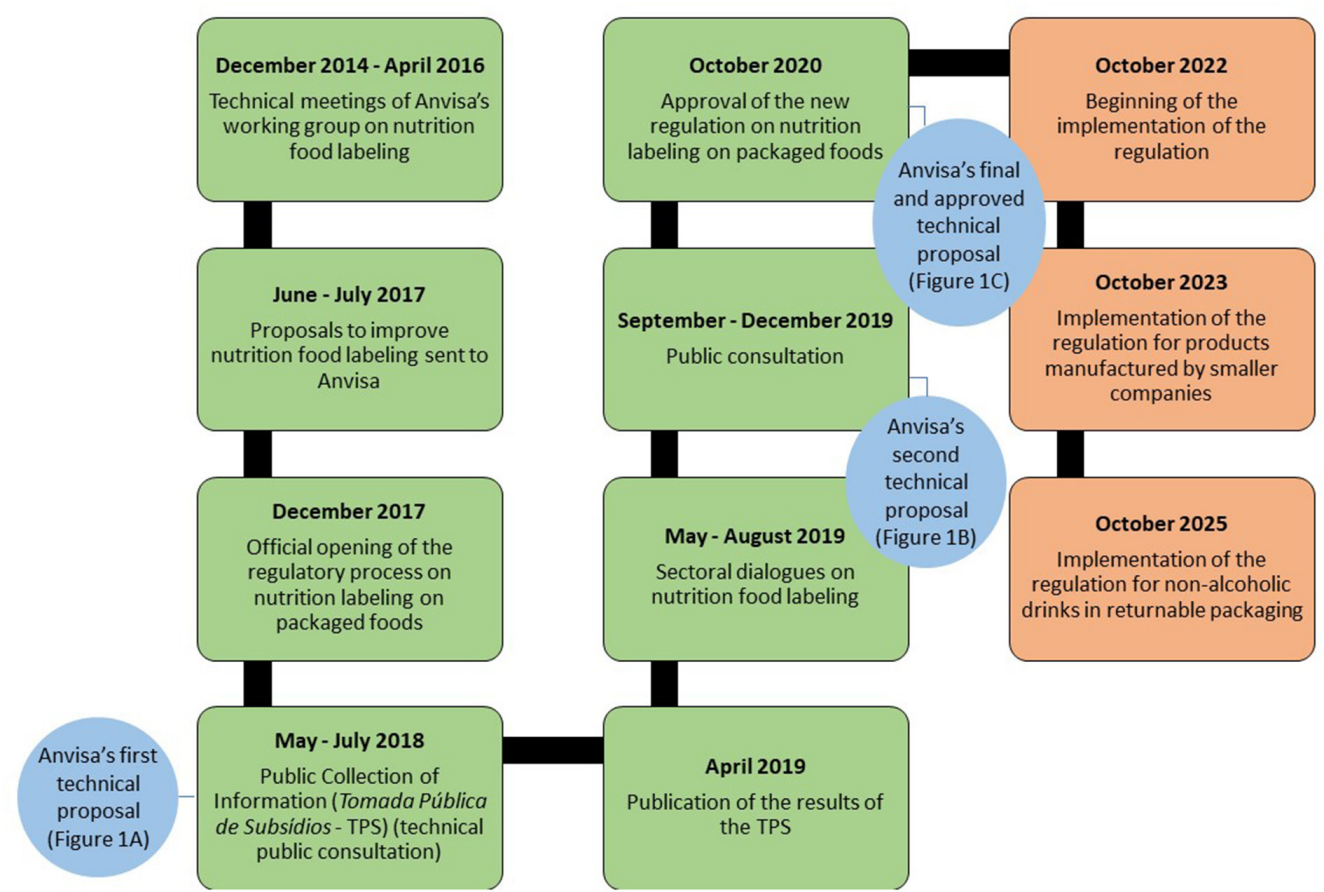
Timeline of the regulatory process of nutrition labeling on packaged foods and its implementation. Brazil, 2014–2025. Boxes in green contemplate events of the regulatory process that have already happened. Boxes in red contemplate events predicted to happen in the next years. Circles in blue represent Anvisa's technical proposals throughout the regulatory process.

It took 6 years for Anvisa to reach a decision and approve the nutrition labeling regulation (Figure 2), due to the organization of the regulatory process, food industry interference and the COVID-19 pandemic. However, its *vacatio legis*^1^ time will take 2 years plus the time of adaptation. Selected products will be allowed a longer implementation period, including foods with returnable packaging such as sugar sweetened beverages, which is among the top most consumed ultra-processed products in Brazil (68) and is associated with increased risk of diabetes and obesity (69). A total of 5 years until the nutrition labeling regulation is fully implemented is a long wait for a policy that is a public health priority. Latin American countries that have approved similar regulations required or will require less time for the new regulations to be implemented than in Brazil (4–8).

## Conclusion

The recently approved nutrition labeling regulation of packaged foods in Brazil reflects a regulatory process that invited technical and scientific contributions from inside and outside Brazil and incorporated evidence-based elements of nutrition disclosure tools. The conflict of interest free scientific evidence that was provided during the regulatory process was not always sufficient to reach public health policy decisions and not all of the recommendations were included in the approved regulation.

An evaluation process is likely to be conducted by Anvisa, which may result in technical improvements of the norm. Despite this government-led evaluation, independent impact evaluation and monitoring shall be conducted by researchers. Impact evaluation studies should target changes in knowledge and purchase behavior of consumers, and product reformulation by the food industry. Evidence from independent and government-led evaluations should be considered in future improvements of the nutrition labeling regulations in Brazil so the effectiveness of the regulation to protect public health and correct unintended setbacks are enhanced.

The newly approved and soon-to-be implemented nutrition labeling regulation in Brazil is certainly good news for the Brazilian population despite the limitations outlined in this commentary. Robust scientific evidence, free of industry influence, and the active participation of civil society and academia in the regulatory process have contributed to also improving the information available on the nutrition facts panel and ensures that relatively unhealthy foods mandatorily display a “high in” FoPNL to highlight nutrients in excess. In doing so, Brazil joins a growing list of countries, especially those in the LAC region, which are implementing a suite of public policies to tackle the burden of malnutrition. This is a step in the right direction, that builds the foundation for continued improvements to the labeling regulations and for the introduction of complementary food policies that restrict marketing to children, ban sales of these unhealthy foods in schools, and increase taxes for the products that receive a FoPNL. Strengthening the collaboration between civil society, the government and academia will be crucial for improving what has already been achieved.

## Author contributions

LAM, CAB, NK, and APBM have been involved in drafting the manuscript and revising it critically for important intellectual content. All authors have made substantial contributions to the conception of the paper and gave final approval of the version to be published.

## Funding

This research was funded by Bloomberg Philanthropies, Grant Number BRAZIL-RIIO-05B.

## Conflict of interest

The authors declare that the research was conducted in the absence of any commercial or financial relationships that could be construed as a potential conflict of interest.

## Publisher's note

All claims expressed in this article are solely those of the authors and do not necessarily represent those of their affiliated organizations, or those of the publisher, the editors and the reviewers. Any product that may be evaluated in this article, or claim that may be made by its manufacturer, is not guaranteed or endorsed by the publisher.

## Footnotes

^1^Latin expression that means the period between the publication date of a legal norm and the beginning of its validity. In general, the *vacatio legis* is expressed in an article in the end of the law with the following text: “The present law enters into force after (the number of) days of its official publication”.
